# Can counter‐advertising dilute marketing effects of alcohol sponsorship of elite sport: A field experiment

**DOI:** 10.1111/add.16317

**Published:** 2023-08-10

**Authors:** Helen Dixon, Maree Scully, Jeff Niederdeppe, Emily Brennan, Kerry O'Brien, Brian Vandenberg, Simone Pettigrew, Melanie Wakefield

**Affiliations:** ^1^ Cancer Council Victoria Melbourne VIC Australia; ^2^ Melbourne School of Psychological Sciences The University of Melbourne Parkville VIC Australia; ^3^ Jeb E. Brooks School of Public Policy and Department of Communication Cornell University Ithaca NY USA; ^4^ School of Social Sciences Monash University Clayton VIC Australia; ^5^ The George Institute of Global Health Newtown NSW Australia; ^6^ Present address: Australian Institute of Family Studies Southbank VIC Australia

**Keywords:** Alcohol, counter‐advertising, counter‐marketing, field experiment, marketing, sponsorship, sport

## Abstract

**Aims:**

To test whether showing spectators counter‐advertisements exposing alcohol harms alone, or exposing alcohol harms and alcohol sponsorship, before watching an alcohol‐sponsored sporting event promotes less favourable post‐event attitudes and intentions towards alcohol sponsor brands and alcohol in general.

**Design:**

On‐line between‐subjects experiment.

**Setting:**

Australia.

**Participants:**

A sample of Australian adults aged 18–49 years who planned to watch an alcohol‐sponsored National Rugby League (NRL) State of Origin series game was recruited through an online panel.

**Interventions:**

Participants were randomly assigned to one of three counter‐advertising conditions: control (neutral advertisement); counter‐advertisement exposing alcohol harms; and counter‐advertisement exposing alcohol sponsorship and harms, to view at least four times during the week before watching the alcohol‐sponsored sporting event.

**Measurements:**

Participants (*n* = 1932) completed a pre‐test questionnaire a week before the sporting event. Within 4 days of watching the sporting event, participants completed post‐test measures assessing sponsor brand awareness, attitudes and preferences towards the brand, as well as knowledge, attitudes and intentions for alcohol in general (*n* = 1075).

**Findings:**

Compared with the control advertisement, the counter‐advertisement exposing alcohol sponsorship and harms promoted higher (6–13%) awareness of sponsor brands, less favourable attitudes towards sponsor brands and drinking beer, lower purchase intentions for sponsor brands (Cohen's *d* = 0.15, 0.31, 0.27, respectively) and perceived less image‐based similarity and fit between the sporting event and sponsor brands (Cohen's *d* = 0.20 and 0.56). Both counter‐advertisements promoted lower perceptions of the appropriateness of consuming alcohol while watching sport (Cohen's *d* = 0.22 and 0.34), higher awareness of alcohol harms (6–34%) and higher intentions (8–13%) to reduce alcohol consumption than the control advertisement.

**Conclusions:**

At alcohol‐sponsored sporting events, counter‐advertisements addressing alcohol harms may promote knowledge of harms and intentions to drink less. Counter‐advertisements that additionally expose and critique alcohol sponsorship may detract from perceptions of sponsor brand image and intentions to purchase the sponsor's products.

## INTRODUCTION

Alcohol is interwoven in Australian social life and sporting culture despite its many detrimental health, social and economic consequences [1, 2, 3, 4, 5]. Widespread commercial promotion of alcohol through sport contradicts public health efforts to deter people from taking up drinking and promote reduced alcohol consumption among existing drinkers. In Australia, multi‐national alcohol companies invest tens of millions of dollars annually in sponsorship of sporting and cultural events and related promotions [6, 7]. Many of Australia's peak sporting organizations (e.g. Australian Football League (AFL), National Rugby League (NRL), Cricket Australia) have sponsorship arrangements with alcohol brands. More than 7 million Australians attend at least one sporting event a year [8], and an estimated 11.7 million watch sport on commercial free‐to‐air TV every week [9]. Viewers are exposed to higher volumes of alcohol advertising during sports broadcasts than during other programming [10, 11]; nine of the top 10 programmes on free‐to‐air TV in 2021 were sports broadcasts [12]. While sporting event attendance is more common for affluent groups [8], all Australia's major sporting codes [i.e. AFL, NRL, cricket, soccer] broadcast games on free‐to‐air TV, exposing Australians of all socio‐economic positions to alcohol sport sponsorship. Although men are more likely than women to have attended a sporting event in the past year (50% compared to 37%) [8] or watched or listened to sport via mass media in the past week (56% compared to 33%) [13], these figures suggest that a sizeable proportion of women are also regularly exposed to alcohol sport sponsorship.

Sponsorship of high‐profile sport typically involves multiple complementary marketing strategies, such as: advertising at sporting events; logos on uniforms; naming of a series, game or stadium; exclusive product category sale rights at events; commercial break advertisements in broadcasts; product endorsements by sport stars. Sport‐linked social media strategies are used to achieve higher consumer engagement to amplify the connection between alcohol brands and the sport spectator experience [14]. Sport sponsorship provides marketers with a powerful and unique vehicle for building brand capital, creating an emotional bond with the sponsor brand and associating brands with sporting celebrities [15]. Brands can benefit from ‘image transfer’, whereby positive attributes associated with sport (e.g. strength, vitality) transfer to the sponsor brand [16, 17]. Marketing through sponsorship can increase brand awareness, enhance brand image and increase intentions to purchase sponsor brand products [18, 19, 20, 21]. Exposure to alcohol advertising and sponsorship in sport is associated with more positive attitudes to alcohol products and consumption [22, 23, 24, 25, 26].

Potential methods for curbing pro‐alcohol marketing effects of alcohol sponsorship of sport include banning it, replacing it with sponsorship by healthier products or conducting counter‐advertising (CA) campaigns to bolster spectators’ resistance against its persuasive effects. CA campaigns use health communication and advertising strategies to reduce demand for unhealthy products. Two common CA strategies include: (1) conveying the harms of unhealthy products; and (2) exposing their producers’ motives and portraying their marketing activities as manipulative or unethical [27]. CA can help to reframe perceptions of public health issues, reduce consumer demand for unhealthy products and prompt changes in industry marketing practices. CA offers a promising approach for disrupting the marketing of alcohol through sport (and other settings), complementing other public health efforts to prevent alcohol‐related health harms [28].

To maximize effectiveness, Agostinelli & Grube recommended that alcohol CA should employ peripheral cues (e.g. emotional appeals, attractive or credible sources) to engage audiences and logical information to encourage central processing, so that information can be integrated into lasting attitude changes predictive of behaviour [28, 29]. Theory [30, 31] and prior research [32] highlight how social norms and perceptions of acceptable behaviour influence health‐related behaviours. Alcohol marketing typically portrays alcohol use as a normative behaviour associated with desired attributes such as athleticism, success, glamour and independence, which encourages consumption. To counter persuasive impacts of such marketing and help to shift social norms, denormalization of alcohol through CA holds promise. Hammond *et al*. [33] distinguished between social denormalization, which seeks to change the broad social norms around the risky behaviour to shift it to being perceived as an abnormal, undesirable practice, and industry denormalization, which seeks to raise public awareness of industry responsibility for their products’ health harms and expose their manipulative marketing tactics. Shifting the focus from the individual's health behaviour to questioning and denormalizing the supply side offers the prospect of addressing industry activity as the chief structural cause of certain health problems [34].

Most CA campaigns aimed at reducing alcohol intake have addressed alcohol‐related health harms and social denormalization of alcohol use. Systematic reviews have found mixed evidence on the efficacy of such campaigns, with potential campaign effects overshadowed by widespread unrestricted alcohol marketing and pro‐drinking social norms [35, 36]. However, when adequate exposure can be achieved, certain alcohol harm prevention CAs are likely to be more effective than others. A previous study examining 83 existing alcohol harm reduction advertisements with 2174 Australian adult weekly drinkers found that CAs portraying health harms of alcohol using graphic imagery and/or a negative emotional tone were most effective in motivating reduced drinking [37].

Some CA campaigns critiquing the alcohol or ‘junk food’ industries have been conducted in the United States, the United Kingdom and Australia, but no systematic evaluations have been reported [27]. Research from other domains suggest that CA exposing alcohol industry marketing practices has the potential to reframe public perceptions of the industry, bolster resistance to influence by such marketing, detract from pro‐alcohol attitudes and discourage alcohol consumption. A naturalistic trial testing effects of two different CAs exposing ‘junk food’ industry sponsorship of sport found that an anti‐industry CA (critiquing sport sponsorship by the ‘Mr. Bigs of junk food’) detracted from favourable attitudes to unhealthy food sponsor brands, while a negative health effects CA (highlighting the illogical association between healthy sports stars and unhealthy food) promoted reduced preferences for fast food among young adult sports spectators of an elite sporting event with prominent junk food sponsorship [38].

CA campaigns exposing tobacco industry marketing tactics can reduce smoking prevalence and initiation and increase intentions to quit [27, 34]. A naturalistic experiment with cinema audiences found that a movie exposing unethical industry conduct and negative information regarding tobacco (*The Insider*) increased negative views of the tobacco industry and reduced smoking intentions [39]. Elements of previous successful tobacco CA campaigns include: highlighting product health harms; exposing industry manipulation of consumers; appealing to negative emotions such as outrage; encouraging resistance to manipulation; critiquing industry marketing; tailoring campaigns to the target audience; creating a campaign brand; and engaging users in campaign design or implementation [27, 40]. A longitudinal study of adult smokers from four countries (*n* = 9058) found that anti‐industry beliefs independently predicted subsequent quitting behaviours, concluding that tobacco‐industry denormalization themes in CA campaigns may help reduce to tobacco use above and beyond more traditional communications targeting social norms [33].

### Aim

The primary aim of this study was to establish whether showing spectators CA prior to viewing an alcohol‐sponsored elite sporting event bolstered their resistance to the influence of pro‐alcohol sport sponsorship as measured at follow‐up. Two types of CA were tested: a more standard, CA exposing alcohol‐related health and social harms (hereafter referred to as ‘CA‐harms’); and a new CA exposing the coercive intent of alcohol sport sponsorship as well as alcohol‐related health harms (hereafter referred to as ‘CA‐sponsorship’). It was hypothesized that compared to a control condition:

- CA‐harms and CA‐sponsorship would: increase beliefs about the harms of alcohol addressed in the respective CAs (H1); detract from positive attitudes and intentions towards alcohol consumption in general (H2).
- CA‐sponsorship would: heighten awareness of alcohol sponsors (H3); detract from perceptions of imaged‐based similarity between the sponsor brand and the sporting event and event/team‐sponsor fit (H4); diminish attitudes, preferences and purchase intentions for sponsor brands (H5).

## METHOD

### Design

**FIGURE 1:**
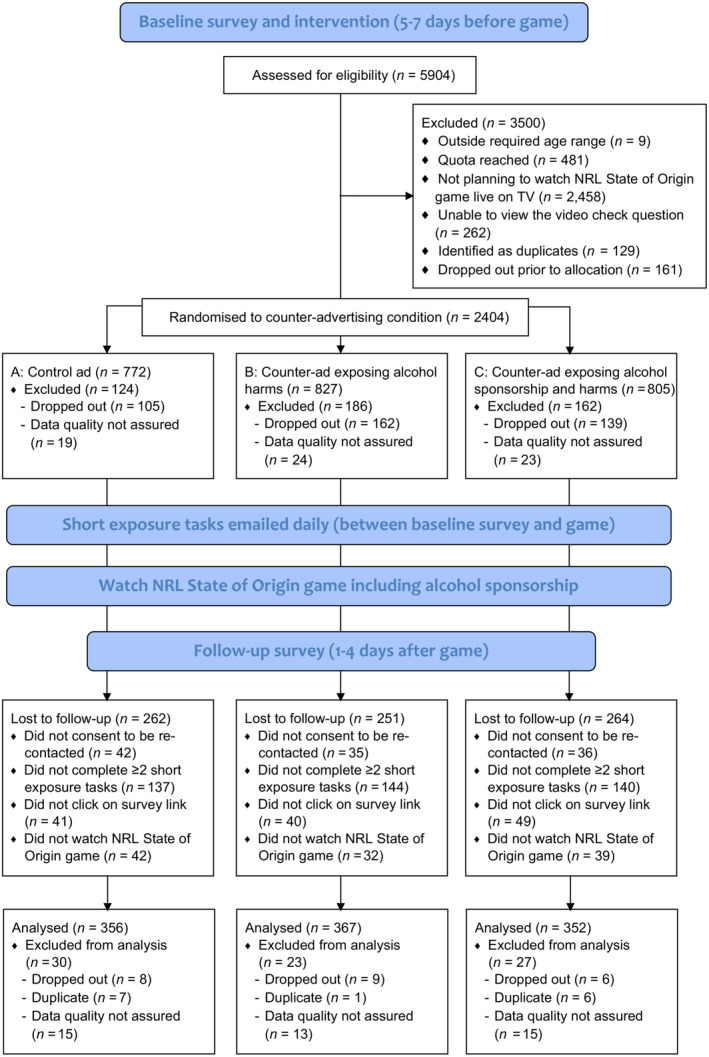
CONSORT flow diagram.

Between‐subjects, web‐based experiment comprising three CA conditions: control (neutral advertisement); CA‐harms; or CA‐sponsorship, which participants viewed in the week leading up to watching an alcohol‐sponsored sporting event. See Figure 1 for the CONSORT diagram.

### Participants

A sample of Australian adults aged 18–49 years who planned to watch an upcoming NRL game with prominent alcohol sponsorship was recruited through Ipsos’ non‐probability national on‐line research panel, with additional sample sourced via its panel partners. To avoid priming or biasing participant responses, the study was described as being about drink products during the recruitment phase, with no references made to sport sponsorship or alcohol marketing. Duplicate participants were identified and removed using digital fingerprint technology. Participants received points for completing each survey (baseline and follow‐up) and short CA exposure task (i.e. viewing their assigned CA in response to a daily e‐mail prompt in the week leading up to the NRL game) which they could redeem for rewards such as shopping gift cards.

We sought to recruit sufficient numbers of male and female sports spectators to enable evaluation of the efficacy of the CAs with both genders. As young males are difficult to recruit via on‐line research panels, quotas were applied to achieve approximately even numbers of males and females, and participants aged 18–34 and 35–49 years, in each condition at baseline. A further screening question assessed participants’ frequency of drinking alcohol over the last 12 months to obtain an approximate 80/20 split of at least monthly drinkers/irregular and non‐drinkers at baseline. To detect effects of a similar magnitude (i.e. Cohen's *d* = 0.22–0.35) to those found in experimental studies testing audience responses to sport sponsorship [21, 41], CA [38, 42] and/or anti‐industry media content [39], we estimated that with a power of 0.80 (*P* < 0.05), a minimum of *n* = 326 participants per condition would be required at follow‐up. Using criteria determined a priori, only those participants who completed the baseline and follow‐up surveys and at least two of the short exposure tasks were included in the final sample.

### Procedure and setting

Participants were randomly assigned to one CA condition (control, CA‐harms or CA‐sponsorship). They completed a baseline questionnaire and viewed their assigned CA in its entirety (30‐sec version) twice, 5–7 days before viewing one of three televised NRL State of Origin matches in June/July 2021. This elite Australian sporting series featured prominent alcohol sponsorship with both competing teams’ player uniforms including an alcohol sponsor logo (XXXX beer for the Queensland Maroons and Tooheys New lager for the NSW Blues). Large, superimposed Victoria Bitter (VB; State of Origin series alcohol sponsor) logos also appeared in both halves of the playing field during the television broadcast. The free‐to‐air TV broadcasts of each of these games attracted an audience of approximately 2 million viewers and were among the top 10 most watched programmes for 2021 [12]. On each of the intervening days between the baseline survey and viewing the event, participants were invited to complete a short exposure task where they viewed their assigned CA again and answered a single rating question after the advertisement had played in full. The task rotated between showing participants either a 15‐ or 30‐sec version of their assigned CA. Within 4 days of watching the event, participants completed follow‐up measures assessing awareness, attitudes and preferences towards alcohol sponsor brands as well as knowledge, attitudes and intentions about alcohol consumption and harm in general. The study protocol was approved by Cancer Council Victoria's Human Research Ethics Committee (IER #2002).

### CA intervention

#### Control

This advertisement, promoting a laptop computer, had nothing to do with alcohol or sport sponsorship.

#### CA‐Harms

This CA (‘Know When to Say When’) was developed by the New South Wales Government, Australia for a campaign that aired in that state in 2011. It vividly depicted short‐term health and social harms of excess alcohol consumption and was ranked among the top 25% for motivating reduced drinking in an experiment testing 83 existing alcohol harm reduction advertisements with Australian adult weekly drinkers [37].

#### CA‐Sponsorship

**FIGURE 2:**
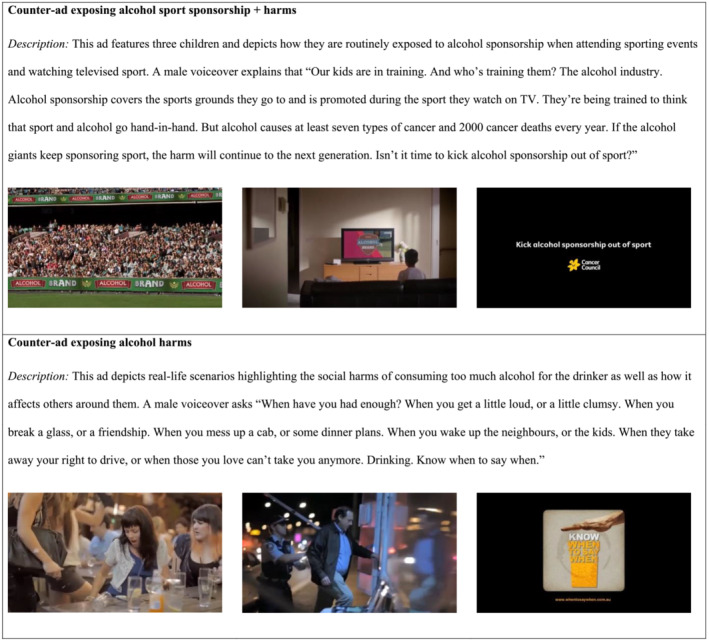
Description of and stills from the advertisements used to represent the two counter‐advertisement conditions.

This CA ('Training') was professionally produced for this study, informed by qualitative research exploring spectator responses to alcohol sport sponsorship. It critiqued alcohol sports sponsorship (without targeting specific brands) and presented one long‐term health harm (cancer). Mixed‐methods testing of four potential concepts [i.e. six focus groups (*n* = 47) with adults aged 18–59 years who watched televised sport at least weekly and individually rated each concept on Likert‐type scales before engaging in a group discussion] indicated that the selected concept had strong potential to draw attention to the manipulative tactics of the alcohol industry, including their use of sport sponsorship to promote their harmful alcohol products to children. See Figure 2 for descriptions and still images of key scenes from the two CAs. All advertisements used in the study were of comparable length (15‐ and 30‐sec versions) and production quality.

### Measures

#### Alcohol harm beliefs

Participants were prompted at follow‐up to report, based on what they know or believe, whether drinking alcohol increases a person's chances of six short‐term alcohol harms (a lot, somewhat, a little, not at all, don't know; each of these harms was addressed in CA‐harms) and is a cause of five long‐term alcohol harms (no, yes, don't know; only cancer was addressed in CA‐sponsorship). Responses were collapsed into a lot versus not for the short‐term alcohol harm items and yes versus no/don't know for the long‐term alcohol harm items.

#### Attitudes towards consuming alcohol while watching sport

At follow‐up, participants recorded their level of agreement (1 = strongly disagree to 7 = strongly agree) with four statements: ‘In Australia, drinking alcohol [when attending a live sporting event/while watching live sport on TV] (like the footy or the cricket) is [normal/acceptable]’. Responses to these four attitudes were averaged to create a summary measure.

#### Alcohol attitudes

Using items adapted from Houben *et al*. [43], participants were asked at both time‐points (i.e. baseline and follow‐up) about their attitude towards drinking beer using six 7‐point semantic differential scales anchored by unpleasant/pleasant, boring/fun, bad/good, risky/safe, unhealthy/healthy and negative/positive. These six attitudes were averaged at each time‐point to form a summary measure.

#### Next week drinking intentions

At follow‐up, participants indicated on a 4‐point ordinal scale (definitely will not, probably will not, probably will, definitely will) how likely it was that they would: avoid drinking alcohol completely; reduce the number of occasions when they drink alcohol; reduce the amount of alcohol they have on each drinking occasion, in the next week. In line with previous studies [44, 45], responses were collapsed into definitely/probably will versus definitely/probably will not. Participants who reported not consuming alcohol in the past 12 months were excluded from these analyses.

#### Brand awareness

Using an item adapted from Jalleh *et al*. [46], participants were prompted at both time‐points to list up to three brands that came to mind when they thought about alcohol. Binary variables were created to indicate if participants listed at least one of the three alcohol sponsor brands (XXXX, Tooheys New and/or Victoria Bitter) at each time‐point.

#### Sponsorship recall and recognition

Following the brand awareness question at follow‐up, participants were asked which sponsors of the NRL State of Origin they recalled, before being presented with a list of 15 alcohol, soft drink and sport/energy drink brands and asked to indicate if they recognized any of them as NRL State of Origin sponsors. Participants who listed/selected at least one of the three alcohol sponsor brands were classified as having unaided sponsorship recall and prompted sponsor recognition, respectively.

#### Image‐based similarity

At the start of the follow‐up survey, participants were prompted to take a moment to think about the NRL State of Origin and how it looked, sounded and felt to watch it on television, and then rate how well (1 = not at all to 7 = very well) eight adjectives (exciting, athletic, healthy, elite, fun, Australian, competitive and tough) described the NRL State of Origin. Later in the follow‐up survey, participants rated how well the same eight adjectives described each of the alcohol sponsor brands. Based on Gwinner & Eaton [47], a summary index of image‐based similarity between the NRL State of Origin and the three alcohol sponsor brands was computed by: summing the absolute differences between participants’ ratings of the NRL State of Origin and each alcohol sponsor brands on the eight adjectives (range = 0–48); reverse coding the summed absolute difference scores so that higher numbers indicated greater image‐based similarity; and averaging the three scores.

#### Event/team‐sponsor fit

At follow‐up, using a scale developed by Speed & Thompson [48], participants indicated their agreement (1 = strongly disagree to 7 = strongly agree) with five statements assessing the fit between: XXXX and the Queensland State of Origin team; Tooheys New and the New South Wales State of Origin team; Victoria Bitter and the NRL State of Origin Series. The five statements included: ‘There is a logical connection between [event/team] and [sponsor brand]’; ‘The image of the [event/team] and the image of [sponsor brand] are similar’; ‘[sponsor brand] and the [event/team] fit together well’; ‘[sponsor brand] and the [event/team] stand for similar things’; and ‘It makes sense to me that [sponsor brand] are sponsoring the [event/team]’ [48]. A summary index of event/team sponsor fit was created by averaging participants’ scores on the three scales.

#### Brand attitudes, preferences and purchase intentions

Using questions adapted from previous sponsorship research [16, 21, 38], at both time‐points participants recorded their attitude (1 = negative to 7 = positive) towards each alcohol sponsor brand, selected which brand they would most prefer to buy if they had to choose between XXXX, Tooheys New, Victoria Bitter (sponsor brands), Carlton Dry and Great Northern Brewing (other beer brands), and indicated how likely (1 = very unlikely to 7 = very likely) they would be to purchase each sponsor brand the next time they have to choose a brand of beer to buy. Summary indices of brand attitudes and purchase intentions were computed by averaging ratings across the three alcohol sponsor brands at each time‐point. Binary variables denoting whether participants selected one of the three sponsor brands as the brand they would most prefer to buy at each time‐point were created.

To minimize response bias and control for order effects, masking questions were included at baseline for outcomes measured at both time‐points and the order of presenting items within question sets was randomized.

### Statistical analysis

Data were analysed using Stata/MP version 16.1 (StataCorp, College Station, TX, USA). To test for differences by condition at follow‐up, logistic (for binary outcomes) and linear (for continuous outcomes) regressions were conducted with the control condition as the reference category. All models controlled for days elapsed between surveys, dose of advertising exposure (based on summed length of assigned CA viewed in baseline survey and completed short exposure tasks; mean = 157.6 sec, SD = 25.6 sec, range = 90–195 sec) and State of Origin game number (1, 2 or 3) as well as baseline responses where the outcome was measured at both time‐points. Adjusted proportions and adjusted means calculated from these regression models are reported. Each model was also re‐run to examine whether there was an interaction between condition and participants’ gender; however, as only one of the 25 interactions tested was statistically significant (a rate below that which would be expected by chance alone), these results are not presented. There were no missing data for any of the outcome measures or control variables. As the analysis was not pre‐registered, results should be considered exploratory.

## RESULTS

### Sample characteristics

A total of 1075 eligible adults completed the baseline and follow‐up surveys and at least two of the short exposure tasks (see Table 1 for a summary of the final sample's characteristics by condition). No differential attrition was observed across conditions, with 44% of cases overall either lost to follow‐up (*n* = 777) or excluded from analysis (*n* = 80). The final sample was comparable to the baseline sample (*n* = 1932) on all characteristics listed in Table 1 (*P* > 0.05). Participants’ dose of advertising exposure was similar across conditions (*F*_(2,1072)_ = 0.71, *P* = 0.492).

**TABLE 1:** Sample characteristics by counter‐advertisement condition (*n* = 1075).

		Counter‐advertisement condition		
	Total (*n* = 1075)	Control advertisement (*n* = 356)	Counter‐advertisement exposing alcohol harms (CA‐harms) (*n* = 367)	Counter‐advertisement exposing alcohol sponsorship and harms (CA‐sponsorship) (*n* = 352)
	%	%	%	%
*Gender*				
Male	52.0	51.7	53.4	50.9
Female	48.0	48.3	46.6	49.1
Age (years)				
18–34	48.7	49.2	47.7	49.4
35–49	51.3	50.8	52.3	50.6
Mean (SD)	34.59 (7.72)	34.24 (7.92)	35.01 (7.67)	34.50 (7.56)
Highest level of education completed				
Non‐tertiary	45.8	44.7	49.3	43.2
Tertiary	54.2	55.3	50.7	56.8
SES (area‐based)^a^				
Low SES	23.5	24.7	22.1	23.7
Medium SES	35.9	35.7	36.5	35.4
High SES	40.6	39.6	41.4	40.9
Parent (any aged child)				
No	32.5	34.6	32.4	30.4
Yes	67.5	65.4	67.6	69.6
Frequency of drinking alcohol over last 12 months				
At least weekly	66.1	66.3	66.5	65.6
At least monthly (but less than weekly)	23.4	22.8	22.6	25.0
Less than monthly/never	10.4	11.0	10.9	9.4

*Note*: Percentages are rounded so may not sum to 100%. All sample characteristics were assessed at baseline.

^a^ SES was determined according to the Australian Bureau of Statistics’ Index of Relative Socio‐Economic Disadvantage ranking for Australia using participants’ home postcodes [57]. Participants who resided in a postcode ranked in the bottom third of the index were categorized as low SES, those in the middle third of the index as medium SES and those in the upper third as high SES. SES information is missing for two participants, as they provided invalid postcodes.

### Effects of CA on alcohol harm beliefs, alcohol attitudes and next week drinking intentions at follow‐up

Table 2 shows that, as hypothesized (H1), participants who saw either CA reported stronger beliefs about the harms of alcohol addressed within the respective CAs compared to the control condition: for short‐term harms addressed in CA‐harms, percentage point differences ranged from 7 to 15%; and for the long‐term health harm (cancer) addressed in CA‐sponsorship (34% higher). Participants who saw CA‐sponsorship also showed increased beliefs about various short‐term harms, plus long‐term harms of heart disease, diabetes and liver disease, that were not addressed within CA‐sponsorship (6–10% higher).

**TABLE 2:** Regression models testing effects of counter‐advertisement condition on alcohol harm beliefs, alcohol attitudes and next week drinking intentions at follow‐up (*n* = 1075).

	Counter‐advertisement condition										
	Control advertisement (*n* = 356)			Counter‐advertisement exposing alcohol harms (CA‐harms) (*n* = 367)				Counter‐advertisement exposing alcohol sponsorship and harms (CA‐sponsorship) (*n* = 352)			
	Adjusted proportion/mean (SE)	AOR/B	95% CI	Adjusted proportion/mean (SE)	AOR/B	95% CI	*P*	Adjusted proportion/mean (SE)	AOR/B	95% CI	*P*
Short‐term alcohol harm beliefs											
Causing serious injury to themselves^CA‐H^	33.8%	Ref		**41.2%**	**1.38**	**(1.02, 1.87)**	**0.038**	**42.2%**	**1.44**	**(1.06, 1.95)**	**0.021**
Causing serious injury to others^CA‐H^	27.0%	Ref		**36.9%**	**1.58**	**(1.15, 2.17)**	**0.005**	**38.8%**	**1.71**	**(1.25, 2.36)**	**0.001**
Getting arrested^CA‐H^	25.4%	Ref		**33.4%**	**1.48**	**(1.07, 2.04)**	**0.018**	**33.9%**	**1.51**	**(1.09, 2.09)**	**0.013**
Upsetting a friend^CA‐H^	24.7%	Ref		**38.5%**	**1.92**	**(1.39, 2.65)**	**< 0.001**	**34.4%**	**1.61**	**(1.16, 2.23)**	**0.005**
Upsetting a family member^CA‐H^	30.7%	Ref		**45.3%**	**1.87**	**(1.38, 2.54)**	**< 0.001**	**39.6%**	**1.48**	**(1.09, 2.02)**	**0.013**
Getting into trouble at work^CA‐H^	32.2%	Ref		**45.9%**	**1.79**	**(1.32, 2.42)**	**< 0.001**	**41.8%**	**1.52**	**(1.11, 2.06)**	**0.008**
Long‐term alcohol harm beliefs											
Cancer^CA‐S^	40.1%	Ref		46.4%	1.29	(0.96, 1.73)	0.090	**73.8%**	**4.22**	**(3.06, 5.80)**	**< 0.001**
Heart disease	65.0%	Ref		69.0%	1.20	(0.88, 1.64)	0.250	**75.2%**	**1.64**	**(1.18, 2.27)**	**0.003**
Diabetes	52.9%	Ref		58.0%	1.23	(0.92, 1.65)	0.167	**63.3%**	**1.54**	**(1.14, 2.09)**	**0.005**
Liver disease	84.0%	Ref		88.9%	1.52	(0.99, 2.35)	0.056	**89.7%**	**1.65**	**(1.06, 2.59)**	**0.028**
Overweight or obesity	82.4%	Ref		86.2%	1.33	(0.89, 2.00)	0.162	86.8%	1.41	(0.93, 2.13)	0.105
Arthritis (distractor item)	18.9%	Ref		22.7%	1.26	(0.88, 1.82)	0.207	21.1%	1.15	(0.79, 1.66)	0.472
Attitudes towards consuming alcohol while watching sport (mean, SE)^a^	5.75 (0.07)	Ref		**5.49 (0.07)**	**−0.26**	**(−0.45, −0.08)**	**0.006**	**5.31 (0.07)**	**−0.45**	**(−0.64, −0.25)**	**< 0.001**
Alcohol attitudes (mean, SE)^b^	4.28 (0.05)	Ref		4.20 (0.05)	−0.08	(−0.22, 0.06)	0.270	**3.81 (0.05)**	**−0.46**	**(−0.61, −0.32)**	**< 0.001**
Next week drinking intentions^c^											
Avoid drinking alcohol completely	34.9%	Ref		37.1%	1.10	(0.80, 1.50)	0.561	**44.3%**	**1.49**	**(1.09, 2.03)**	**0.013**
Reduce number of drinking occasions	50.5%	Ref		**58.9%**	**1.41**	**(1.04, 1.90)**	**0.027**	**60.5%**	**1.50**	**(1.11, 2.04)**	**0.009**
Reduce amount consumed per drinking occasion	48.4%	Ref		**58.9%**	**1.53**	**(1.13, 2.06)**	**0.006**	**61.6%**	**1.71**	**(1.26, 2.33)**	**0.001**

*Note:* Regression models controlled for days elapsed between surveys, dose of advertising exposure, game number and where measured, baseline responses. Bold‐type results are significant at *P* < 0.05.

Abbreviations: AOR, adjusted odds ratio; B, unstandardised regression coefficient; CA‐H, harm addressed in CA‐harms; CA‐S, harm addressed in CA‐sponsorship; CI, confidence interval; Ref, referent category in regression model; SE, standard error.

^a^ Participants’ attitudes towards consuming alcohol while watching sport (four statements; Cronbach's α = 0.90) were recorded on a 7‐point scale (1 = strongly disagree to 7 = strongly agree).

^b^ Participants’ alcohol attitudes were recorded on 7‐point semantic differential scales (unpleasant/pleasant, boring/fun, bad/good, risky/safe, unhealthy/healthy, negative/positive; Cronbach's *α* = 0.90).

^c^ Excludes participants who reported not consuming alcohol in the past 12 months (*n* = 52).

Compared to the control condition, those who saw CA‐harms or CA‐sponsorship held less favourable attitudes towards consuming alcohol while watching sport (Cohen's *d* = 0.22 and 0.34, respectively). Attitudes towards alcohol (beer) were less favourable for those who viewed CA‐sponsorship (Cohen's *d* = 0.31), but not CA‐harms. Participants exposed to either CA were more likely to intend to reduce their number of drinking occasions and the amount of alcohol they have per drinking occasion in the next week than participants exposed to the control advertisement (8–13% higher). Those exposed to CA‐sponsorship were more likely to intend to avoid drinking alcohol completely in the next week (9% higher; see Table 2). Findings provide support for H2 in relation to CA‐sponsorship but are somewhat mixed for CA‐harms.

### Effects of CA on alcohol sponsor brand awareness, attitudes, preferences and intentions at follow‐up

Consistent with H3, participants exposed to CA‐sponsorship had higher unprompted awareness of any alcohol sponsor brand and were more likely to recall or recognize at least one of the NRL State of Origin alcohol sponsor brands compared to the control condition (6–13% higher; see Table 3). Those exposed to CA‐sponsorship perceived lower image‐based similarity between the alcohol sponsor brands and the NRL State of Origin series and weaker fit between the event/team and the alcohol sponsor brands than the control condition (Cohen's *d* = 0.20 and 0.56, respectively, supporting H4). In line with H5, participants exposed to CA‐sponsorship showed less favourable attitudes and weaker purchase intentions towards the alcohol sponsor brands (Cohen's *d* = 0.15 and 0.27, respectively) compared to the control condition. Contrary to H5, participants who viewed CA‐sponsorship did not show significantly reduced odds of preferring alcohol sponsor brands. Those who saw CA‐harms showed no significant differences to the control condition on any of the alcohol sponsor brand outcomes (see Table 3).

**TABLE 3:** Regression models testing effects of counter‐advertisement condition on alcohol sponsor brand awareness, attitudes, preferences and intentions at follow‐up (*n* = 1075).

	Counter‐advertisement condition										
	Control advertisement (*n* = 356)			Counter‐advertisement exposing alcohol harms (CA‐harms) (*n* = 367)				Counter‐advertisement exposing alcohol sponsorship and harms (CA‐sponsorship) (*n* = 352)			
	Adjusted proportion/mean (SE)	AOR/B	95% CI	Adjusted proportion/mean (SE)	AOR/B	95% CI	*P*	Adjusted proportion/mean (SE)	AOR/B	95% CI	*P*
Brand awareness	52.3%	Ref		55.3%	1.16	(0.84, 1.62)	0.366	**63.1%**	**1.74**	**(1.24, 2.44)**	**0.001**
Unaided sponsorship recall	43.1%	Ref		47.4%	1.19	(0.89, 1.59)	0.251	**56.2%**	**1.69**	**(1.26, 2.28)**	**0.001**
Prompted sponsor recognition	79.9%	Ref		80.6%	1.05	(0.73, 1.52)	0.794	**86.3%**	**1.60**	**(1.07, 2.39)**	**0.022**
Image‐based similarity (mean, SE)^a^	30.68 (0.51)	Ref		31.43 (0.51)	0.74	(−0.67, 2.16)	0.303	**28.64 (0.52)**	**−2.04**	**(−3.47, −0.61)**	**0.005**
Event/team‐sponsor fit (mean, SE)^b^	4.50 (0.07)	Ref		4.49 (0.07)	−0.01	(−0.21, 0.19)	0.918	**3.74 (0.07)**	**−0.76**	**(−0.96, −0.56)**	**< 0.001**
Brand attitudes (mean, SE)^c^	4.10 (0.05)	Ref		4.12 (0.05)	0.02	(−0.12, 0.16)	0.785	**3.95 (0.05)**	**−0.15**	**(−0.29, −0.00)**	**0.043**
Brand preferences	50.9%	Ref		47.4%	0.79	(0.54, 1.16)	0.224	48.2%	0.83	(0.57, 1.22)	0.349
Brand purchase intentions (mean, SE)^d^	3.51 (0.05)	Ref		3.42 (0.05)	−0.09	(−0.24, 0.06)	0.231	**3.08 (0.05)**	**−0.43**	**(−0.58, −0.28)**	**< 0.001**

*Note:* Regression models controlled for days elapsed between surveys, dose of advertising exposure, game number and where measured, baseline responses. Bold‐type results are significant at *P* < 0.05.

Abbreviations: AOR, adjusted odds ratio; B, unstandardized regression coefficient; CI, confidence interval; Ref, referent category in regression model; SE, standard error.

^a^ Sum of the absolute differences in participants’ ratings of the National Rugby League (NRL) State of Origin series and the three alcohol sponsor brands on eight adjectives (Cronbach's *α*s ranged from 0.88 to 0.89 for each brand). Scores have been reversed such that higher numbers indicate greater image‐based similarity between the NRL State of Origin and the three alcohol sponsor brands (range of scores = 0–48).

^b^ Participants’ perceptions of event/team–sponsor fit (five statements; Cronbach's αs ranged from 0.94 to 0.95 for each brand) were recorded on a 7‐point scale (1 = strongly disagree to 7 = strongly agree).

^c^ Participants’ brand attitudes were recorded on a 7‐point semantic differential scale (1 = negative to 7 = positive).

^d^ Participants’ purchase intentions were recorded on a 7‐point scale (1 = very unlikely to 7 = very likely).

## DISCUSSION

Pervasive alcohol sponsorship of elite sport is a powerful marketing strategy that promotes alcohol brands and consumption [15, 26]. Guided by theory and prior research on CA [27, 28], this field experiment tested the efficacy of using CA strategies to bolster spectators’ resistance to pro‐alcohol marketing effects of prominent alcohol sponsorship of an elite sporting event, the Australian NRL State of Origin Series. Findings indicate that CA can offset the persuasive influence of prominent alcohol sponsorship of elite sport, but that different types of CA messaging have differing impacts. In line with previous research on industry denormalization [32, 33] in the domain of junk food [37], a CA exposing the coercive intent of alcohol sport sponsorship (CA‐sponsorship) heightened spectators’ awareness of sponsor brands but tainted perceptions of sponsors’ brand image and reduced purchase intentions for sponsors’ products. This CA also addressed health harms (link between alcohol and cancer), whereas the messaging of the other CA tested (CA‐harms) centred on social denormalization and health harms (alcohol's short‐term health and social harms) [33, 34]. in line with previous research attesting to the effectiveness of CAs portraying alcohol harms using a negative emotional tone [37], both CAs successfully promoted knowledge of alcohol health harms and intentions to drink less. A previous study also found that the industry denormalization CA was also effective at increasing spectators’ support for policies aimed at restricting sports‐related alcohol marketing [49]. Collectively, findings suggest that CAs that direct viewers to look ‘upstream’ at systemic contributors to ‘downstream’ health problems (such as industry marketing and sport sponsorship) can help to garner public support for policy change and promote beliefs, attitudes and intentions conducive to reduced personal consumption of harmful products.

### Effects of CA on beliefs, attitudes and intentions towards alcohol in general

Both CAs incorporated design elements informed by theories of social norms [30, 31] and the elaboration likelihood model of persuasion [29]. Each CA proved effective in promoting more negative perceptions and intentions towards alcohol in general, including more negative perceptions of the appropriateness of consuming alcohol while watching sport. As well as promoting stronger beliefs about the particular harms addressed in the respective CAs, CA‐sponsorship promoted stronger beliefs about additional short‐ and long‐term harms, suggesting that this CA primed viewers to think about other health and social harms associated with alcohol beyond cancer. CA‐sponsorship not only detracted from perceptions of specific sponsor brands, but also of alcohol in general, resulting in intentions to drink less or abstain from drinking in the next week. Findings support Agostinelli & Grube's contention that CA offers a potentially promising approach for disrupting alcohol marketing, reframing perceptions about the place of alcohol in society and reducing consumer demand for alcohol [28]. It is possible that CA strategies could be extended to combatting additional forms of alcohol marketing beyond sports sponsorship, as CA has proved effective in diminishing persuasive impacts of front‐of‐pack promotions and TV product advertisements for unhealthy foods [50].

Based on the elaboration likelihood model of persuasion [29] and previous research assessing impacts of public health campaigns [35], we would expect CAs to initially impact cognitive and affective variables after relatively brief exposure, as used in this study (on average, ~2.5 minutes in total), and ultimately impact behavioural variables following more intensive, cumulative exposure as in a ‘real‐world’ CA campaign. Findings demonstrate that brief exposure to alcohol CA can disrupt beliefs and attitudes known to predispose consumers to alcohol consumption, plus drinking intentions, suggesting that more intensive exposure could affect distal variables such as drinking behaviour. Further research could test this by exposing participants to a more intensive alcohol CA campaign and using behavioural response measures such as alcohol purchasing or consumption.

### Effects of CA on awareness, attitudes and preference for alcohol sponsor brands

We expected that the messaging of CA‐sponsorship (exposing and critiquing alcohol sports sponsorship) would make spectators more consciously attentive to alcohol sponsors of the sporting event. Accordingly, CA‐sponsorship increased awareness of sponsor brands (H3), whereas CA‐harms (which did not address sponsorship) did not. Importantly, the increased awareness of sponsor brands promoted by CA‐sponsorship came at a significant cost to brand image. In the days following the sporting event, spectators who had seen CA‐sponsorship showed less favourable attitudes towards alcohol sponsor brands, perceived less of a fit between the sporting event and the alcohol sponsors, perceived less similarity between the image of the sporting event and the alcohol sponsors (H3–4) and held weaker intentions to purchase alcohol sponsor brands (supporting H5). In general, the process of image transfer that ordinarily occurs when spectators are exposed to sponsor brands paired with elite sports stars and events [16, 17] appears to have been disrupted by CA‐sponsorship. However, neither CA promoted a reduction in preference for sponsor brands (contrary to H5), suggesting that brand loyalty was harder to derail. As per the elaboration likelihood model of persuasion [28, 29], a possible mechanism for this overall pattern of findings could be that the arguments and evidence presented in CA‐sponsorship led spectators to view alcohol sponsors more deliberatively (central route to persuasion), eroding sport sponsorship's usual power to harness peripheral persuasive cues (e.g. emotionally charged event; attractive sports stars) to achieve image transfer from the sporting event to the sponsor brands. If, as this theory predicts, attitude change achieved via the central route to persuasion is more robust than that achieved via the peripheral route, it would be interesting for future research to examine how long attitude changes promoted by CAs persist.

### Strengths and limitations

In this experiment, participants (most of whom were drinkers) had relatively brief exposure to a single exemplar of two types of CA and small to medium effect sizes were found some days after exposure. The strongest effects occurred in response to CA‐sponsorship (e.g. 34% increase in awareness of the link between alcohol and cancer; 9–13% increases in various intentions to drink less in the next week). Such effect sizes are typical for media interventions [51, 52], but because they have vast population penetration can translate into clinically meaningful population effects.

While it is encouraging that both CAs promoted reduced drinking‐related intentions, such self‐report measures can be subject to socially desirable responding [53] and an intentions–behaviour gap [54]. Consequently, we cannot be certain that observed effects of the CAs on intentions translate into reductions in drinking behaviour in the community. Future research could explore these issues in relation to alcohol CA campaigns (e.g. using check measures to detect socially desirable responding; assessing drinking behaviour as a primary outcome).

Theory and prior research (e.g. [27, 40]) informed the design and production of CA‐sponsorship and selection of CA‐harms for testing, as they were expected to be engaging and persuasive. Different executions of these two types of CA might have promoted different responses. CA‐harms was broadcast in one state of Australia more than a decade ago and can be accessed via YouTube, whereas CA‐sponsorship was produced for this study. Thus, we cannot rule out the possibility that familiarity with CA‐harms may have limited the impact of its exposures in this study context.

We tested these CAs’ ability to diminish promotional effects of alcohol sports sponsorship in an elite male‐only team sport where the main alcohol sponsors were beer brands, and effects were found for adult male and female sports spectators. Future research could test whether alcohol CA is also effective in detracting from the appeal of alcohol sponsors with younger spectators who are not yet drinkers, or combatting effects of different forms of alcohol marketing disseminated through other settings or media.

Study strengths were that we tested professionally produced, broadcast quality CAs that pre‐tested well with the target audience. We also timed the experiment around an actual sporting event with prominent alcohol sponsorship and spectators as participants. Given that we found clear effects of our short‐term CA intervention (on average, six exposures to a single ≤ 30‐sec CA, totalling approximately 2.5 minutes) relative to prolific alcohol sponsorship promoted throughout the NRL State of Origin series, it seems likely that an alcohol CA campaign with a stronger media presence could have a meaningful impact. Nonetheless, in the absence of restrictions on alcohol marketing, industry‐funded marketing campaigns will continue to outspend and overshadow even well‐funded alcohol CA campaigns [35, 36].

Ultimately, complete removal of harmful product advertising (e.g. through regulation) should be more effective than CA in protecting the public from exposure and influence by such advertising [50]. However, in contexts where lucrative, long‐running sponsorship deals between sporting entities and alcohol brands persist, CA offers the next best strategy for diminishing persuasive impacts of alcohol sponsorship. The challenge for organizations designing and implementing real‐world CA campaigns will be to gain access to media channels with high potential to reach and impact the target audience, when such media outlets may also be invested in airing advertising promoting the very products that CA campaigns are trying to ‘un‐sell’. One way of overcoming this challenge is to mandate CA appearing alongside harmful product advertising, as in the case of the United States’ former ‘fairness doctrine’ period for tobacco advertising [55], and the recent requirement in Australia that mass media advertisements for on‐line wagering products must be followed by a tagline exposing risks of gambling such as ‘Chances are you're about to lose’ [56].

## CONCLUSIONS

Counter‐advertisements addressing alcohol harms can promote knowledge of harms and intentions to drink less. Counter‐advertisements that additionally expose and critique alcohol sponsorship can detract from perceptions of sponsor brand image and intentions to purchase sponsor's products. In contexts where alcohol sports sponsorship occurs, CA has potential utility for diminishing marketing impacts of alcohol sponsorship. This approach could be scaled‐up for mass media dissemination in the lead‐up to major alcohol sponsored sporting events, providing public health organizations and policymakers with an important tool for helping to protect spectators against the known, persuasive effects of pervasive alcohol sport sponsorship.

## AUTHOR CONTRIBUTIONS

**Helen Dixon:** Conceptualization (lead); formal analysis (equal); funding acquisition (lead); investigation (lead); methodology (lead); project administration (lead); visualization (equal); writing—original draft (lead); writing—review and editing (lead). **Maree Scully:** Data curation (lead); formal analysis (lead); funding acquisition (supporting); investigation (equal); methodology (equal); project administration (lead); visualization (equal); writing—original draft (equal); writing—review and editing (equal). **Jeff Niederdeppe:** Formal analysis (supporting); funding acquisition (supporting); investigation (supporting); methodology (supporting); writing—original draft (supporting); writing—review and editing (supporting). **Emily Brennan:** Funding acquisition (supporting); investigation (supporting); methodology (supporting); writing—original draft (supporting); writing—review and editing (supporting). **Kerry O'Brien:** Funding acquisition (supporting); investigation (supporting); methodology (supporting); writing—original draft (supporting); writing—review and editing (supporting). **Brian Vandenberg:** Funding acquisition (supporting); investigation (supporting); methodology (supporting); writing—original draft (supporting); writing—review and editing (supporting). **Simone Pettigrew:** Funding acquisition (supporting); investigation (supporting); methodology (supporting); writing—original draft (supporting); writing—review and editing (supporting). **Melanie Wakefield:** Funding acquisition (equal); investigation (supporting); methodology (supporting); project administration (supporting); supervision (lead); writing—original draft (supporting); writing—review and editing (supporting).

## DECLARATION OF INTERESTS

Authors (H.D., M.S., E.B. and M.W.) are employed by a non‐profit organization that conducts research, public health interventions and advocacy aimed at reducing alcohol‐related health harms in the community, especially those pertaining to cancer. The other authors (J.N., K.O., B.V., S.P.) have no conflicts of interest to declare.

## Data Availability

The data that support the findings of this study are available from the corresponding author upon reasonable request.
